# DNA methylation biomarkers in asthma and rhinitis: Are we there yet?

**DOI:** 10.1002/clt2.12131

**Published:** 2022-03-11

**Authors:** Evangelia Legaki, Christos Arsenis, Styliani Taka, Nikolaos G. Papadopoulos

**Affiliations:** ^1^ Allergy and Clinical Immunology Unit Second Pediatric Clinic National and Kapodistrian University of Athens Athens Greece

**Keywords:** allergic respiratory diseases, allergic rhinitis, asthma, DNA methylation, allergische atemwegserkrankungen, allergischer schnupfen, asthma, DNA‐methylierung, epigenetik

## Abstract

The study of epigenetics has improved our understanding of mechanisms underpinning gene‐environment interactions and is providing new insights in the pathophysiology of respiratory allergic diseases. We reviewed the literature on DNA methylation patterns across different tissues in asthma and/or rhinitis and attempted to elucidate differentially methylated loci that could be used to characterize asthma or rhinitis. Although nasal and bronchial epithelia are similar in their histological structure and cellular composition, genetic and epigenetic regulation may differ across tissues. Advanced methods have enabled comprehensive, high‐throughput methylation profiling of different tissues (bronchial or nasal epithelial cells, whole blood or isolated mononuclear cells), in subjects with respiratory conditions, aiming to elucidate gene regulation mechanisms and identify new biomarkers. Several genes and CpGs have been suggested as asthma biomarkers, though research on allergic rhinitis is still lacking. The most common differentially methylated loci presented in both blood and nasal samples are ACOT7, EPX, KCNH2, SIGLEC8, TNIK, FOXP1, ATPAF2, ZNF862, ADORA3, ARID3A, IL5RA, METRNL and ZFPM1. Overall, there is substantial variation among studies, (i.e. sample sizes, age groups and disease phenotype). Greater variability of analysis method detailed phenotypic characterization and age stratification should be taken into account in future studies.

## 1 INTRODUCTION

Respiratory diseases associated with allergy, such as asthma and rhinitis, constitute a major and continuously growing public health concern specially in the western world. Notably, all atopies combined, now affect approximately 20% of the global population.^1^ Asthma is unanimously accepted as an important socioeconomic issue that affects roughly 300 million people. Allergic rhinitis (AR) shows an ever‐increasing prevalence on a global scale with more than 600 million patients.^2^ A considerable percentage present severe morbidity which medication fails to control, leading in a sharp decrease of their life quality. These statistics are bound to increase and rhinitis along with asthma appear as major epidemics of current time and rapid initiatives need to be taken towards the direction of prevention, therapeutics and disease management. Evidence indicates that etiology of asthma and allergic diseases is complex and has strong genetic and environmental components. Since the epigenome is modified by environmental factors, epigenomic and transcriptomic profiling may provide added value for individual prediction models of asthma outcomes, in addition to genomic profiling.^3^

Epigenetic mechanisms provide a new understanding of gene‐environment interactions. Modifications to the epigenome mediate endogenous or exogenous environmental exposures on immune development.^4^ In mammals, DNA methylation regulates gene expression and ensures genome stability. Methylation almost exclusively occurs in the context of the CpG dinucleotide. In promoters and other *cis*‐regulatory sequences (i.e. enhancers, insulators) DNA methylation may hamper transcription factor binding, further contributing to gene silencing.^5^ During fetal life and after birth, DNA methylation continues to play a pivotal role in cellular commitment and differentiation.^6^, ^7^, ^8^ Besides these programmed changes, gain and loss of DNA methylation occur in various genomic regions as a consequence of cellular and environmental stresses and stochastic changes during lifetime. Because the respiratory system is commonly exposed to environmental stimuli (chemicals, dust, bacteria, viruses, etc.), the epigenome of the airway cells is prone to dynamic changes that may, ultimately, affect gene expression. DNA methylation can be assessed either over the entire genome (whole‐genome methylation profiling) or by candidate studies designed to search specifically for differentially methylated regions (DMRs) or CpGs in specific genes or regions in the DNA.^3^

## 2 AGE‐DEPENDENT EPIGENETIC CHANGES

One of the most prominent external factors influencing DNA methylation changes is aging and it has been reported that the chronological age can be determined by DNA methylation patterns.^9^ Aberrant DNAm level of aging‐related genes has been recorded in asthma patients.^10^ Accelerated epigenetic aging, meaning the difference between epigenetic age and chronological age, has been associated with a large number of disease and an overall greater risk of death^9^ while longevity has been associated with decelerated epigenetic aging.^11^ The development of allergic diseases is likely age‐dependent during childhood^12^; however, chronological age alone does not fully explain disease variability. Epigenetic aging has now also been assessed in the context of atopic or allergen sensitization and asthma using a variety of different clocks.^13^, ^14^ There are several methods available to estimate epigenetic aging,^9^, ^15^, ^16^, ^17^, ^18^ and among them, the Horvath method for epigenetic age estimation (DNAmAge) is used widely and has shown high accuracy.^19^ Data have shown a significant epigenetic age acceleration in children with current asthma (0.74 years) and even greater age acceleration for children with allergic asthma (1.30 years). For every 10‐fold increase in FeNO, the epigenetic age was accelerated by 1.11 years. In total, epigenetic age of nasal samples is accelerated by asthma and is correlated with elevated biomarkers of allergic disease such as IgE and FeNO.^13^ Furthermore, accelerated epigenetic aging in children at 7–8 years of age was associated with increased serum IgE levels and a 1.2‐ to 1.3‐fold increased risk of atopic sensitization, or sensitization to environmental or food allergens for every year increase in epigenetic age.^14^

Machine learning approaches are increasingly used to address healthcare problems; up to date, only one study has been conducted to predict lung functions using machine learning approaches by utilizing the effect of DNAmAge and accelerating age on lung function. Arefeen et al.^20^ suggested that apart from the previously described factors height, weight, and sex, changes in epigenetic age acceleration between 10 and 18 years can improve the prediction of FEV1 and FVC at 18 years of age and proposed five selected regression models for machine learning techniques to be used for lung function prediction.

DNA methylation patterns are tissue specific, and one critical limitation for human epigenetic studies is that tissues that are relevant for disease etiology cannot be easily obtained from patients and study participants.^21^ Various biological specimens have been used to analyze DNA methylation in airway diseases such as sputum, bronchoalveolar lavage (BAL) and blood samples. Overall, nasal and bronchial pseudostratified epithelia are similar in their histological structure and cellular composition^5^; however, genetic and epigenetic regulation may differ across tissues.

A literature review was conducted in PubMed database for articles published up to August 2021. The search terms used were ‘asthma’ or ‘allergic asthma’ or ‘allergic rhinitis’ or ‘allergic respiratory diseases’ and ‘epigenetics’ or ‘DNA methylation’ or ‘epigenome wide association study’. The identified studies were divided into categories regarding the type of tissue used, that is, bronchial epithelial cells, nasal epithelial cells and blood cells. This review aims to describe DNA methylation patterns across different tissues which are associated with allergic respiratory diseases such as asthma and rhinitis. Using a state‐of‐the‐art perspective, including the concept of epigenetic aging and machine learning, we try to elucidate differentially methylated loci that could be used as immune age biomarkers.

## 3 DIFFERENTIAL DNA METHYLATION IN BRONCHIAL EPITHELIAL CELLS

Over the last decade, 11 relevant studies were identified, which assessed the methylation status of the genome in asthmatic bronchial epithelial cells (BECs), the primary cell type exposed to inhalants, and the corresponding effect on gene expression (Table 1). BECs are the primary cell type exposed to inhalants, but their location makes collection more technically challenging compared to nasal cells, hence less studies are inclined to include them. Nevertheless, several CpGs were identified as having an altered methylation status, along with various DMRs, both when comparing asthmatics to controls, as well as different asthma subgroups to each other. Although not always, this difference in methylation was often found to directly or indirectly affect proximal or distal gene expression levels. Various sample sizes were employed, however, most had *n* ≤ 25, and only three were larger with *n* > 50. Apart from two studies that included children, most focused on adults, roughly between their twenties and forties, and used the Illumina 450k Beadchip, on par with the rest of the epigenome field.

**TABLE 1:** DNA methylation studies in bronchial epithelial cells

Study population	Ethnicity‐origin	Age average (range) or: mean ± SD/SEM	Gender (male%)	Hospital‐ or population‐based cohort	Asthma/AR diagnosis	Sampling method	Methylation measurement method	Important findings	Reference
DNA methylation cohort: *n* = 25 (7 controls, 9 atopic, 4 atopic asthmatics, 5 non‐atopic asthmatics)	N/A	DNA methylation cohort: Control 7.28 (4.6–10.1) Atopic 6.78 (4.5–10.9); Atopic asthmatic 8.4 (3.3–14.6); Non‐atopic asthmatic 5.96 (2.4–10.5)	N/A	Hospital	Asthma: Physician‐diagnosed plus documented wheeze by a physician in the past 12 months. Positive responses to ISAAC and ATS questionnaires.	Bronchial brushings	Illumina GoldenGate methylation cancer Panel I (Illumina)	Asthmatics versus controls: no difference in methylation signature non‐asthmatic atopics versus controls: no difference in methylation signature atopic asthmatics versus non‐atopic asthmatics: no difference in methylation signature	Stefanowicz et al. (2012)^22^
Gene expression cohort: *n* = 44 (15 controls, 14 atopic, 15 atopic asthmatics)		Gene expression cohort: Control 5.07 (1.2–12.9; Atopic 7.86 (2.2–16.5); Atopic asthmatic 7.74 (1.3–14.1)			Atopy: Positive RAST or skin prick tests to common allergens.			Asthmatics versus atopic: 8 CpGs, 8 genes: CRIP1, FGFR1, STAT5A, S100A2, ITGA2, EGR4, ID1, IGSF4C, functions include: Cell adhesion, mitogenesis, differentiation, cell cycle progression, senescence, cell growth and proliferation. Gene expression (asthmatics): ↓: STAT5, ↑: CRIP1	
*n* = 24 (7 controls, 10 atopic, 7 non‐atopic asthmatics to dust mites)	N/A	Control 50 (22–71), asthmatic atopic 35 (21–60), asthmatic non‐atopic 47 (28–78)	Control: 14.3% asthmatic; atopic: 40% asthmatic; non‐atopic: 14.3%	Hospital	Asthma diagnosis by physicians, met global initiative for asthma (GINA) asthma definition. History of dyspnea and wheezing during last 12 months, and one of: (1) >15% increase in forced expiratory volume in 1 s (FEV1) or >12% increase + 200 ml following inhalation of a short‐acting bronchodilator; (2) <10 mg/ml PC20 methacholine; (3) >20% increase in FEV1 after 2 weeks inhaled/systemic corticosteroid treatment. Atopy: Wheal reaction ≥3 mm diameter of skin prick tests, or ≥ than histamine induced		Human methylation27 Beadchip (Illumina)	Asthmatics versus controls: 1 CpG, 1 gene: LCN6, function: Involved in single fertilization; Atopic versus non‐atopic: 24 CpGs, 53 DMRs, 52 genes (most significant: MAP3K5, CDH1, C11orf47, B3GALT1, PKHD1, LOC63928, AKR1C4, LRTM1, KRTAP17‐1, CASP1, MGC33407), associated with multicellular process, response to organic substance, hormone metabolic process, and growth factor receptor binding.	Kim et al. (2013)^23^
Cultured cells: *n* = 116 (58 controls, 58 IL‐13‐treated)	Freshly isolated cells: African American = 69; European American = 42; Other = 5	Cultured cells: donors 45 ± 13 SD	Asthma 29% control 33%	Population	Asthma: doctor's diagnosis, no conflicting pulmonary diagnoses.	Cultured cells: Lung explants	Infinium human methylation 450K Bead Chip (Illumina)	Cultured cells: IL‐13 treated versus controls: 6522 CpG‐sites, 3771 genes: Notably TNXB (associated with multiple CpGs), CHI3L1 (asthma biomarker), POSTN & SERPINB2 (markers of Th2‐high asthma phenotype), overal significantly enriched for genes associated with asthma. Gene expression: ↓:48% ↑:52%	Nicodemus‐Johnson et al. (2016)^24^
Freshly isolated cells: *n* = 118 (41 controls, 77 asthmatic); atopy (%) = 63 controls, 88 asthma		Freshly isolated cells: asthma = 39.25 ± 12.95 SD; control = 37.56 ± 11.35 SD				Freshly isolated cells: Bronchial brushing		Freshly isolated cells: asthmatics versus controls: 2020 CpG‐sites (31% overlap with cultured cell CpGs); 74% methylation effect direction overlap	
*n* = 24 (13 controls, 11 asthmatics)	N/A	20.7 (8–42)	Asthma 45%; control 46%	Hospital	N/A	Bronchial brushings, pronase digestion	N/A	Asthmatics versus controls: gene expression: 6 genes: ↓: AURKA (kinase), DZIP3 (ligase), EHMT2 and SUV39H1 (methyltransferases), ↑: CREBBP and EP300 (acetyltransferases); protein expression: AURKA ↑ in asthmatics (but only AURKA & CREBBP examined)	Stefanowicz et al. (2017)^25^
*n* = 115 (41 controls, 74 asthmatics)	African American = 69; European American = 42; Other = 5	Asthma = 39.09 ± 12.94 SD; control = 37.56 ± 11.35 SD	Asthma 30%; control 33%	Population	Asthma: doctor's diagnosis, no conflicting pulmonary diagnoses, were using medication (75% inhaled corticosteroids, 41% oral corticosteroids, 4% smokers)	Bronchial brushing	Infinium human methylation 450K Bead Chip (Illumina)	Asthmatics versus controls: 40,892 CpGs, notable genes: CCL26 (chemokine elevated in asthmatic airways), MUC5AC (mucin with roles in airway defence)	Nicodemus‐Johnson et al. (2016b)^26^
								Gene expression: Modest correlation with nearest gene expression	
N/A	N/A	N/A	N/A	N/A	N/A	N/A	Sodium bisulfite sequencingPyrosequencing: EpiTect bisulfite kit (Qiagen)	Gene expression (17q12‐q21 genes): unaffected: ORMDL3, IKZF3 ↑: GSDMB ↑↑: GSDMA, ZPBP2	Moussette et al. (2017)^27^
								Found DNA methylation changes resulted in allelic bias changes of ZPBP2 and ORMDL3	
*n* = 33 (pronase digestion: nine controls, eight asthmatics/bronchial brushings: seven controls, nine asthmatics)	N/A	Pronase digestion asthmatic 18.63 ± 3.173 SEM Pronase digestion non‐asthmatic 21 ± 3.27 SEM Bronchial brush asthmatic 31.85 ± 5.0 SEM Bronchial brush Non‐asthmatic 56.22 ± 3.8 SEM	Pronase digestion asthmatic 25%; Pronase digestion non‐asthmatic 78%; Bronchial brush asthmatic 44%; Bronchial brush non‐asthmatic 71%	Hospital	N/A	Bronchial brushings, pronase digestion	Infinium human methylation 450K Bead Chip (Illumina)	DNA methylation profiles alter based on isolation method.	Clifford et al. (2019)^28^
							Bisulfite PCR‐pyrosequencing	Control pronase versus control bronchial brush: 111 CpGs, 103 genes: Including CHRNE, EDAR, GALNT9, LOC149837, LINC00654, GRIK2, CECR1, OR2I1P, DAXX, HEYL.	
								Asthmatics pronase versus controls pronase: 15 DMRs, 1 gene: DUSP22 (asthma‐associated) asthmatics bronchial brush versus controls bronchial brush: 849 DMRs, genes: Notable KALRN and WNT7B (asthma‐associated)	
								No DMRs were identified by both pronase and bronchial brushings	
*n* = 135 (70 controls, 26 persistent asthmatics, 39 remission asthmatics)	N/A	Controls = 39.5 ± 2.03 SEM persistent asthmatics = 48.8 ± 1.85 SEM remission asthmatics = 47.5 ± 2.33 SEM	Persistent asthmatics 58%; remission asthmatics 46%; controls 57%	Population	Asthma: Documented reversibility and/or airway hyperresponsiveness to histamine (PC 20 =< 32 mg/ml).	Bronchial brushings	Infinium human methylation 450K Bead Chip (Illumina)	Asthma versus remission: 4 CpGs, 42 DMRs, genes: Notable KRBOX1, TNXB, LBX1, DGKQ, RPL13 A (role in chronic inflammation amelioration)	Vermeulen et al. (2020)^29^
					Clinical remission: no asthma attack/wheeze in last 3 years, and no asthma medication.			Gene expression: ↑: ACKR2, DGKQ, RPL13 A.	
					Complete remission: no airway hyperresponsiveness to histamine & AMP (>32 mg/ml in 30 s tidal breathing and >320 mg/ml in 2 min tidal breathing), no airflow obstruction signs (FEV 1% predicted >80% pre‐bronchodilator or >90% post bronchodilator).			Remission versus controls: 1163 CpGs, 328 DMRs, genes: Notable PDLIM4, ETV6, GMPR	
					Persistent asthma: Divided on the basis of use of inhaled corticosteroids				
*n* = 13 (5 control, 8 asthmatics)	White	Controls: 37.35 ± 13.26; asthmatics: 44.53 ± 13.55	Asthma 37.5%; control 40%	Hospital	Asthma diagnosis by pulmonologist/allergologist as per GINA guidelines. 37.5% of asthmatics were atopic	Bronchial brushings	Illumina Infinium EPIC array	Asthmatics versus controls: Methylation Asthmatics: ↑ cytoskeletal remodeling and cell growth: FIGN, PIK3R5, DSC1, TEKT1, PCDHB11, DLC1, TMDO3, TNXB, CAPNSZ, RBM38 ↑ ion transport and metabolism: PDZK1, DDO, HPD, ATP11 B, MANBA, UROS ↑ T‐cell signaling pathway: CMIP ↓ pro‐inflammatory cytokines: IL‐6R, IL1R1, IL1R2, IL36 B, IL17 B, IL17RE, IL4I1 ↑ regulatory genes: IL10RA, TGFBR2 ↑ chemokines: CCL26, CCL24 ↑ bronchial barrier regulation: AMOTL1, CLDN11, CLDN18, MAGI1, TJP2, JAM3 (tight junction family members)/ACTB (actin protein)/TUBA1C, ROCK2, LLGL1 (cytoskeleton components)	Wawrzyniak et al. (2021)^30^
								Methylation Controls: ↑ transcription coactivation, posttranscription activation, intracellular signaling: GREBBD, SORBS2, PCBR3, TBX2, BRD2, DAXX, ACTB, POLD2, MGAT3	
								Notable: TET1 (epigenetic modifier) methylated, PRMTs (histone methyltransferases) not methylated	

Notably, two of the three largest studies thus far, were conducted by the same research group.^24^, ^26^ The first looked into the epigenetic response of cultured BECs to interleukin 13 (IL‐13), a key cytokine involved in asthma pathogenesis,^31^ and identified in the IL‐13‐treated cells an epigenetic fingerprint consisting of 6522 differentially methylated CpG sites (44% hypermethylated, 56% hypomethylated) compared to controls, most of which (77%) were near a gene body (41%), or gene transcription start site (36%), totaling 3771 genes. Some genes were associated with multiple of these CpGs, with the authors singling out Tenascin B (TNXB), a member of the tenascin family of extracellular matrix glycoproteins with anti‐adherence effects, due to the presence of 12 hypomethylated CpGs, as well as Chitinase 3‐like 1 (CHI3L1), an asthma biomarker.^32^ Gene expression analysis revealed extensive transcriptomic changes, with 63% of assessed genes (8524/13,532) being differentially expressed (52% increase, 48% decrease), with the authors singling out Chemokine (C‐C motif) ligand 26 (CCL26), a chemokine elevated in asthmatic airways, as well as the T helper 2‐high asthma biomarkers Periostin (POSTN) and Serpin Family B Member 2 (SERPINB2).^33^, ^34^ Interestingly, 21% of genes within 1500 kb of an assayed CpG site were in or near a minimum of one IL‐13‐responsive CpG site, and were significantly enriched for genes associated with asthma. When the methylation status of the 6522 previously identified IL‐13‐responsive CpG sites were subsequently assessed in freshly isolated cells from asthmatics, 31% were found to also be differentially methylated compared to controls, 74% of which had the same direction of methylation effect compared to the cell culture model. Lastly, a weighted gene co‐expression network analysis identified two clusters of highly correlated genes which correlated with clinical phenotypes of either asthma severity and lung function or eosinophilia. These results suggest that part of the epigenetic variation seen in asthmatics may be induced by IL‐13, via persistent methylation changes in asthmatic airways.

The same group subsequently published a study^26^ which presented a full analysis of the comparison between asthmatics and controls from mostly the same population which they previously used to compare the results with their IL‐13 in vitro model. They identified 40,892 differentially methylated CpG‐sites in asthmatics (54% hypermethylated, 46% hypomethylated), and gene expression analysis showed the DMRs modestly correlated with their nearest gene expression, including asthma‐associated genes, with the authors singling out the previously mentioned CCL26, and Mucin 5AC (MUC5AC), with roles in airway defense against particulates/pathogens. Furthermore, a linear model framework showed 9.89% among all CpGs within 5kb of a SNP and 11.96% of DMRs were associated with at least one methylation quantitative trait locus.

Out of the remaining studies comparing asthmatics to controls, one study found no difference in methylation levels,^22^ and another measured similar overall methylation levels, with the only difference being a CpG motif in the promoter of Lipocalin 6 (LCN6), which is involved in male fertility. The rest found significant differences, with a study identifying 864 DMRs associated with 428 genes,^28^ including the following asthma‐associated genes with the greatest effect: Dual Specificity Phosphatase 22 (DUSP22), with roles such as regulation of cell proliferation and migration, Kalirin RhoGEF Kinase (KALRN), with roles such as nervous system development and axon guidance, and Wnt Family Member 7B (WNT7B) of the Wnt signaling pathway. Another study did not directly measure methylation changes, but targeted epigenetic modifying enzymes instead and assessed expression of 82 genes across 5 families(30), with linear regression showing reduced mRNA expression of the kinase Aurora Kinase A (AURKA), the ligase DZIP3, the methyltransferases Euchromatic Histone Lysine Methyltransferase 2 (EHMT2) and Suppressor Of Variegation three to nine Homolog 1 (SUV39H1), as well as increased mRNA expression of the acetyltransferases CREB Binding Protein (CREBBP) and E1A Binding Protein P300 (EP300). Notably, regarding genes involved in epigenetic processes, Tet Methylcytosine Dioxygenase 1 (TET1), which is involved in DNA demethylation, was found to be methylated in another study which used in air‐liquid interface cultures of asthmatic BECs,^30^ whereas Protein Arginine Methyltransferase 1 (PRMT1), a histone methyltransferase, was not. The same study also found higher global methylation levels in asthmatic BECs, and their list of the top 100 group of highly methylated genes includes the previously mentioned LCN6, CCL26, CREBBP and TNXB, with several of the rest of the genes associated with cytoskeletal remodeling, cell growth, ion transport, metabolism, T‐cell signaling pathway, and bronchial barrier regulation (Table 1).

When comparing atopic to non‐atopic asthmatics, one study found no difference between the methylation signatures,^22^ however when it compared non‐asthmatic atopic children to asthmatic it did find 8 differentially methylated CpG sites associated with 8 genes: Early Growth Response 4 (EGR4), S100 Calcium Binding Protein A2 (S100A2), Inhibitor Of DNA Binding 1, HLH Protein (ID1), Cell Adhesion Molecule 4 (CADM4), Cysteine Rich Protein 1 (CRIP1; expression increased in asthmatics), Fibroblast Growth Factor Receptor 1 (FGFR1), Signal Transducer And Activator Of Transcription 5A (STAT5A; expression decreased in asthmatics), Integrin Subunit Alpha 2 (ITGA2), whose functions include cell adhesion, mitogenesis, differentiation, cell cycle progression, senescence, cell growth and proliferation. The other study found six DMRs in six genes hypermethylated among atopic asthmatics, whereas 47 DMRs in 46 genes were hypomethylated,^23^ with a gene ontology analysis associating these 52 genes with the categories: multicellular process, response to organic substance, hormone metabolic process, and growth factor receptor binding.

The third, and most recent, larger study compared persistent asthma and remission (subjects that had not had an asthma attack or wheeze in the last 3 years, and did not use asthma medication), and identified a different methylation profile between them.^29^ Four differentially methylated CpGs and 42 DMRs were identified, including cg08364654, which had 6% lower methylation in remission and was associated with Atypical Chemokine Receptor 2 (ACKR2) expression, cg00741675, which had 11% lower methylation in remission, and was located within and associated with expression of Diacylglycerol Kinase Theta (DGKQ), cg23805470, which resides in but is not associated with the expression of TNXB (also identified by the first large study,^24^ and the second most significant DMR which was associated with Ribosomal Protein L13a (RPL13 A) expression, with a role in chronic inflammation amelioration. When comparing remission to controls, 1163 CpG sites and 328 DMRs were identified as differentially methylated, with around one‐third of CpGs and 20% of DMRs being more methylated in remission. Out of the top 10 CpGs, with the exception of PDZ and LIM Domain 4 (PDLIM4), ETS Variant Transcription Factor 6 (ETV6), Guanosine Monophosphate Reductase (GMPR), they did not correlate with the expression of their nearby genes, but did so with several dozen distant genes. Interestingly, unlike other studies, it examined the cell type composition differences between groups and showed that it can have significant impact to the results.

Two of the studies produced results relating to 17q12–21, the most replicated asthma‐related locus. Firstly, Nicodemus‐Johnson et al.^26^ utilized an advanced omics approach to identify an asthma regulatory locus, outside the linkage disequilibrium block of 17q12–21, which correlated specifically with the expression of ORMDL Sphingolipid Biosynthesis Regulator 3 (ORMDL3; located in 17q12–21), whose associated functions include sphingolipid homeostasis, myelination, ceramide metabolic process, neutrophil degranulation, and smooth muscle contraction. The second relevant study by Moussette et al.^27^ utilized an immortalized human BEC line to examine the effects of a DNA demethylation agent on the expression of five genes of 17q12–q21. Out of those genes, ORMDL3 and IKAROS Family Zinc Finger 3 (IKZF3) expression was unaffected, Gasdermin B (GSDMB) expression increased, whereas Gasdermin A (GSDMA) and Zona Pellucida Binding Protein 2 (ZPBP2) expression were highly upregulated. DNA methylation assays showed ORMDL3 promoter hypomethylation, decrease in ZPBP2 promoter methylation from 16% to 5%, decrease in GSDMA promoter methylation from 57% to 25%, with GSDMA‐CG1 promoter methylation reduced from 9% to 0% (previously found to be hypomethylated in asthmatic females.^35^ The study also demonstrated how changes in DNA methylation could affect allele expression ratios, specifically finding changes in allelic bias of ZPBP2 and ORMDL3.

Overall, although the findings between the studies were not highly comparable, a somewhat common finding was the differential DNA methylation of epithelial barrier genes responsible for functions including adhesion and immune response regulation, as well as genes responsible for cell proliferation, migration and differentiation, along with several epigenetic modifier genes all of which is relevant in asthma, since the condition is characterized by airway hyperresponsiveness, airway wall remodeling and airway inflammation.

## 4 DNA METHYLATION IN NASAL EPITHELIAL CELLS (NECS)

Cells of the nasal epithelium have properties resembling bronchial epithelial cells and nasal brushing is much less invasive than bronchial brushing or BAL; thus, this technique represents a good surrogate model for lower respiratory tract studies.^36^, ^37^ There is evidence that the study of the nasal methylome allows for making reliable conclusions about DNA methylation in the lungs.^38^, ^39^ Furthermore, evidence show that the bronchial epithelium and blood are twice as distant as the bronchial and nasal epithelium, emphasizing that DNA methylation in blood samples may not be informative enough to draw conclusions about methylation marks in the airway.^38^ In the current decade, quite a few nasal methylome studies have been conducted elucidating the complex molecular patterns involved in asthma. In total, methylome studies present a great variability in demographic (age and gender distribution) and clinical characteristics (disease definition, medication, acute infection, smoking, pet exposure); however, it is noteworthy that none of the studies distinguishes subgroup of asthma and/or AR (Table 2). Although, most studies do not include direct association to specific clinical characteristics, few of them have reduced results' variation through adjustment corrections of major components (age, gender).^40^, ^45^, ^46^, ^47^ Four studies directly examined the effect of smoke on DNA methylation. Zhang et al.^46^ and Qi et al.,^50^ did not find any association to second‐hand smoking but Yang et al.^47^ identified 48 DMRs that are significantly associated with environmental tobacco smoke (ETS). Furthermore, Zhu et al.^49^ 2019 showed that DMPs which are associated to asthma severity are also associated to second‐hand smoking.

**TABLE 2:** DNA methylation studies in nasal epithelial cells

Study population	Ethnicity‐origin	Age [average (range), or: mean ± SD/SEM]	Gender (male%)	Hospital OR population‐based cohort	Asthma/AR diagnosis	Sampling method	Methylation measurement method	Important findings	Reference
35 asthmatic children‐ non controls	Caucasian	8.9	77%	Population	Physiciandiagnosis of asthma; reported wheezing symptoms in the previous 12 months; chesttightness and/or use of bronchodilators in the last 12 months	Nasal cell brushing‐Inferior turbinate	PCR‐pyrosequencing on bisulfite‐treated DNA	↓ DNA methylation of the *IL‐6* and *iNOS* promoters ‐ ↑ FENO	Baccarelli et al. (2012)^40^
								Alu and LINE‐1 methylation showed no associations with FENO and FEV1	
Discovery: *N* = 15 asthmatic	33.3% Caucasian	13.4 (7.4–18.0)	73.3%	Hospital	Respiratory symptom score (based on frequency of wheeze, cough, shortness of breath, and chest tightness) and the age‐specific asthma control Test™ scorewas collected	Nasal mucosa brushing‐ epithelial cells (CytoSoft Brush)	Targeted PCR‐pyrosequencing on differentially expressed genes between good and poor responders	Altered DNA methylation at VNN1 promoter‐ VNN1 as a biomarker for corticosteroid treatment	Xiao et al. (2015)^41^
Replication *N* = 25 asthmatics	8% Caucasian	8.1 (5.0–15.1)	64%	Hospital					
*N* = 20 exacerbating asthmatics‐	20% White 70% Black 10% biracial	8.0 (6.0–15.0)	80%	Hospital	Respiratory symptom score (based on frequency of wheeze, cough, shortness of breath, and chest tightness) and the age‐specific asthma control Test™ score was collected	Nasal mucosal brushing‐ epithelial cells (CytoSoft Brush)	Llumina Infinium HumanMethylation450 BeadChip (Illumina)	182 CpGs with common SNPs overlap (*LDHC*, *DNHD1* and *PRRC1)* 127 CpGs without SNP co‐localization (*GALNTL4*, *SRD5A3*, *MED12L‐GPR87* locus and *CARD14)* ↓ methylation of OTX2 gene after treatment	Zhang et al. (2017)^42^
*N* = 13 more exacerbating asthmatics	15%white77%black8%biracial	6 (5.0–8.0)	46%	Hospital					
Population 1 *N* = 10 asthmatics (atopic) *N* = 7 healthy controls	Multiracial	23 (±2.4); 26 (±5.9)	Asthmatics 40%; Healthy 14.3%	Population	Individuals with a history of asthma. Determination of additional clinical characteristics: Airway hyperreactivity, lung function and atopic status via methacholine challenge, spirometry and allergen skin test respectively	Nasal Scrapings of the medial inferior surface of the inferior turbinate after local vasoconstriction and anesthesia	Pyrosequencing global DNA methylation (Alu elements)	↑ global methylation in response to virus infection	McErlaean et al. (2014)^43^
Population 2 N = 6 asthmatics (atopic) *N* = 6 healthy controls		20 (0.9); 31 (9.8	Asthmatics 33.4% Healthy 84%				Infinium human methylation 450 K bead chip microarray (Illumina)	SNORA12 different methylation between atopic asthmatic and healthy subjects	
*N* = 10 asthmatic children *N* = 10 healthy children (ALLIANCE study)	N/A	11.6 (8–15) – full term birth	50%	N/A	Asthma diagnosis based on the 2014 global initiative for asthma (GINA) guidelines	Nasal brushing (3 nm IDB‐G brush)	Infinium human methylation 450 K bead chip microarray (Illumina)	HRVI mediated methylation changes to: AGPAT1, BAT3, NEU1, ANAPC11, MGST3, CCT6A, MICB, SMN1, LOC442454, KLHL8, SLC16A3, GP1BA, DNAJC7, VDAC2, FBXO7, TP53I3	Pech et al. (2018)^44^
							Confirmation study: Pyrosequencing		
Discovery N = 35 asthma‐controls sibling pairs (exposure Sibling Study)	African‐American	11.0 (9.0–14.0)	54%	Mixed	Asthma diagnosis was obtained from the parental report, and confirmed via electronic medical record. (Research electronic data capture‐ REDCap software characterized asthma onset, diagnosis, symptoms, severity, quality of life, medication, environmental exposures, social histories and residential address for the first year of life and for the past 5 years.	Nasal epithelial cell or nasal mucosa sampling with a CytoSoft Brush	Infinium HumanMethylation450 BeadChips (*N* = 12); Validation via Pyrosequencing (*N* = 35)	↓ DNA methylation at TET 1 promoter	Somineni et al. (2016)^45^
Replication *N* = 158 asthmatics *N* = 28 non asthmatics		12.0 (8.0–15.0)	52%		Asthma diagnosis according to American Thoracic Society criteria	Saliva	Replication via Pyrosequencing (*N* = 186)		
Discovery *N* = 54 asthma‐ controls sibling pairs (exposure Sibling Study)	African‐American	12.01 (5–18) Asthmatics; 11.35 (5–18) controls	Asthmatics 63%; Non‐asthmatics 43%	Mixed	Asthma diagnosis was obtained from the parental report, and confirmed via electronic medical record. (Research electronic data capture‐ REDCap software characterized asthma onset, diagnosis, symptoms, severity, quality of life, medication, environmental exposures, social histories and residential address for the first year of life and for the past 5 years:	Nasal epithelial cell or nasal mucosa sampling with a CytoSoft Brush	Infinium HumanMethylation450 BeadChips (*N* = 58); Validation pyrosequencing (*N* = 108)	6 CpGs associated with asthma(no statistical significance) *↓3 non‐SNP CpGs replicated in independent cohots* associated to TET1, *OR2B11* and *NLRP3*	Zhang et al. (2018)^46^
Discovery study (inner city Consortium) *N* = 36 cases with persistent asthma *N* = 36 healthy controls without atopy or asthma (shared controls)	African American 91.7%; Hispanic Latino 8.3%	11 (10–12)	Asthmatics 52.8% Controls 47.2%	Population	(1) physician diagnosis; (2) persistent or uncontrolled disease defined by the National asthma Education and Prevention Program; (3) physiologic evidence of asthma (FEV1 < 85% or FEV1/FVC ratio<85%; and bronchodilator responsiveness (≥12%) or PC20 < 8 mg/ml of methacholine); (4) Positive skin prick test to at least one of a panel of indoor aeroallergensAtopy assessed by positive skin prick testing for multiple allergens or RAST/Phadiatop tests.	Nasal epithelial cells brushing from the posterior portion of the inferior turbinate	Illumina's Infinium human methylation 450k BeadChip	119 genome‐wide significant DMRs associated with 118 unique genes 118 single CpG motifs (DMPs) associated with 107 unique genes: Some gene associated to asthma: ALOX15, CAPN14, HNMT, POSTN Extracellular matrix: COL16A1, COL5A2, COL5A3, ELN, HAS3, MMP14, Immunity: IFNGR2, HLKA‐DPA1, LAG3, NFIL3, PRF1, TNFSF13 Cell adhesion CTNND1, EPPK1, GJA4 epigenetic regulation ATXN7L1, H1F0, HIST1H1D, METTL1 Airway obstruction GABRG3, Obesity C1QTNF1, GPC4 Autophagy AMBRA1	Yang et al. (2017)^47^
Validation 1 N = 30 asthmatics N = 36 shared controls from discovery study	African American	11 (10–12)	N/A				Pyrosequencing ‐targeted genes		
Validation 2 N = 12 asthmatics and *N* = 12 controls without asthma.	Caucasian	24–74	N/A		(1) FEV1 > 55% at visit 1; (2) positive methacholine challenge (PC20 < 12 mg/ml) within last 6 months or demonstrated at visit 1, OR demonstrates improvement in FEV1 of >12% and 200 ml; (3) no history of life threatening asthma; (4) no treatment with bronchial thermoplasty; (5) must be taking inhaled corticosteroid for at least 4 weeks prior to Visit 1		Illumina's Infinium human methylation 450k BeadChip		
EVA‐PR study N = 312 atopic N = 171 non atopic	Hispanic Latino	9–20		Population	Atopy: At least one positive specific IgE.	Nasal specimens from above the inferior turbinate (administration of lidocaine 1%)	EWAS‐Illumina's Infinium human methylation 450k BeadChip	8664 CpGs differentially methylated by atopy, significant CpGs related to epithelial barrier function and immune regulation *CDHR3, CDH26*, SLC9A3, PCSK6, FBXL7, and NTRK1. 1570 CpG‐gene expression pairs were revealed, including 11 of the top 30 EWAS results	Forno et al. (2019)^48^
Internal Validation *N* = 40 atopic asthma *N* = 40 non atopic non‐asthmatics					Atopic asthma: Parental report of having physician‐diagnosed asthma and at least one episode of wheeze in the previous year, plus at least one positive specific IgE		TWAS‐ RNA seq Illumina NextSeq 500 platform Targeted (15 genes) Pyrosequencing		
External Validation (PIAMA) *N* = 207 atopics N = 255 non atopics	American NonHispanic‐ white	16.4(±0.2) atopics 16.3(±0.2) non atopics	Atopics 55.6% Non atopics 43.6%	N/A	Serum specific IgE to the common aeroallergens.	Nasal brushing from lateral area underneath the right inferior turbinate	Illumina's Infinium human methylation 450k BeadChip		
					Sensitization: Specific IgE≥ 0·35 kUA/l. Atopy: Sensitization to any of these allergens. Asthma: Parental “yes” answers to: 1 doctor diagnosis of asthma 2. Wheeze in the last 12 months 3. medication for respiratory or lung problems				
547 children: Atopy and controls (ProjectViva)	Multiracial	12.9 (11.9–15.3)	50.6%	Hospital	Current asthma: mother's report of a doctor's diagnosis of asthma since birth reported on the early teen questionnaire plus report of wheeze or asthma medication in the past year at early teen follow‐up.	Nasal swabs from the anterior nares	EWAS‐ Illumina Infinium HumanMethylation450 BeadChips	Overlapping DMRs across phenotypes.More DMRS for FENO • Th2 activation and eosinophilia (EPX, IL4, IL13, PRG2, CLC, ZFMP1) • Solute carriers and intracellular transport genes (SLC25A25, SLC39A4, DNAH17, VTI1A) • T cell activation (LAX) • Oxidative stress (VKORC1L1) • Mucin production (GALNT7). • Gap junction protein gene (GJA4) • 53% replicated CpGs “inner city Consortium” • 61% replicated CpGs “EVA‐PR” studies	Cardenas et al. (2019)^13^
					Current allergic rhinitis: mother's report of a doctor's diagnosis of hay fever since birth reported on the early teen questionnaire plus report of sneezing, runny nose, or blocked nose without cold or flu in the past year and current symptoms (moderate‐level nasal congestion/stuffiness, nasal blockage, or trouble breathing through the nose in the past month) at the time of nasal swab.				
*N* = 33 non severe asthma N = 22 severe asthma from exposure Sibling study (ESS) and the genomics of secondhand‐smoke exposure in Pediatric Asthma study (GSEP)	African American (54/55)	Non severe asthma: 12.6 (2.4) Severe asthma 13.7 (3.0)	Non severe asthma: 48.5% Severe asthma: 45.5%	N/A	Asthma severity was defined by symptom frequency using previously validated respiratory symptom score (maximum respiratory symptom score, maxRSS) severe asthma was defined as a symptom score of 3 or 4, while non‐severe asthma was defined as a symptom score of 0, 1 or 2. Allergic children were defined as having a positive doctor's diagnosis of allergy.	Nasal epithelial cell or nasal mucosa sampling with a CytoSoft Brush	Illumina Infinium HumanMethylation450 BeadChip (Illumina) (29 ESS participants) Illumina Infinium MethylationEPIC array (33 GSEP participants)	Differential DNAm were observed between non‐severe and severe. 816 DMPs and 10 DMRs associated with asthma severity. 16 pathways were significantly enriched among the 398 genes associated with these DMPs. 59genes with multiple DMPS that is, LTB4R2, DPP10, IL17RA, CYFIP2, DNAH5, MRPL28, and PTPRN2, TMEM51, WDR25, HIPK3, and KLF11 39 DMP levels associated with mRNA levels	Zhu et al. (2020)^49^
Discovery: *N* = 455 children (PIAMA cohort)	Non Hispanic white	16.3 ± 0.2	47.7%	N/A	Asthma: Presence of at least two‐thirds criteria: (1) doctor‐diagnosed asthma ever, (2) wheeze in the past 12 months, and (3) prescription of asthma medication in the past 12 months	Nasal brushing from lateral area underneath the inferior turbinate with Cytosoft brush after local anesthesia with 1% lidocaine	Infinium HumanMethylation450 BeadChips RNA‐seq‐ Illumina HiSeq2500 platform	81 CpG sites associated with rhinitis 75 were associated with AsRh(mixed asthma/rhinitis phenotype) 8 CpG sites associated with all 3 asthma/rhinitis/asthma and‐or rhinitis including NCF2, NTRK1, GJA4, CYP27B1, and ANO1. 20 CpG site–gene pairs: PCSK6, FBXL7 (F‐box and leucine rich and CISH	Qi et al. (2020)^50^
					Rhinitis: Presence of sneezing or a runny or stuffed nose without having a cold in the previous 12 months or the presence of hay fever in the previous 12 months. AsRh: Presence of either asthma or rhinitis				
*N* = 219 asthmatics *N* = 236 healthy controls (EVA‐PR study)	Hispanic Latino	9–20	N/A	Population	Atopy: 1 positive IgE (≥0.35 IU/ml) to five common allergens in Puerto Rico.	Nasal specimens from above the inferior turbinate (administration of lidocaine 1%)	HumanMethylation450 BeadChips	eQTM enriched in pathways for immune processes and epithelial integrity eQTM methylation‐gene pairs (biologically plausible for lung disease and allergy): *PAX8* *ECHDC3*, *LSP1* *HLA‐DQB1* *FRG1B* *KANSL1* STAT4, IL32, STAT1, CCR5, CCL5, HLA‐DMB, HLA‐DMA, CXCR6	Kim et al. (2020)^51^
					Asthma: physician's diagnosis plus at least one episode of wheeze in the previous year				

Among the first potential biomarkers in nasal epithelial cells proposed for asthma was *TET1* as the hypomethylation of its promoter was associated with childhood asthma in African Americans.^45^ Of note, the methylation level of this CpG site was highly correlated across nasal cells, PBMCs and saliva, making it a potential cross‐tissue biomarker for childhood asthma. Notwithstanding, *TET1* has been found up‐methylated in BECS from asthmatics compared to controls. *TET1* encodes a dioxygenase that consecutively converts 5‐methylcytosine (5 mC) into 5‐hydroxymethylcytosine (5hmC), 5‐formylcytosine (5 fC) and 5‐carboxylcytosine (5caC), thus playing a key role in active DNA demethylation and resulting in transcriptional activation of downstream genes such as VEGFA, which is known to be associated with lung function,^52^ particulate matter exposure and asthma.^45^, ^53^, ^54^, ^55^ Apart from *TET1,* the same group later in the Exposure Sibling Study (ESS) identified other two differentially methylated—non SNP CpG sites (cg00112952 and cg14830002) within the promoter region of *OR2B11*, also located approximately 4000 bp downstream the transcription end site of *NLRP3*, which consist a biological plausible regulator of inflammation, immune response and neutrophilic asthma.^46^

Another pioneer study of DNA methylation in nasal cells from asthmatics (*n* = 35, Caucasian children population) showed that FeNO (fractional exhaled nitric oxide) is negatively associated with IL6 and iNOs methylation. While global methylation has been found higher in BECs of asthmatics,^30^ Alu and LINE1‐global methylation was not linked to FeNO and FEV1 (Forced Expiratory Volume) in nasal epithelial cells (NECs). Decreased methylation of IL6 promoter was associated with higher FENO as shown in multivariable regression in an independent fashion of the iNOS promoter status. Nasal cell LINE‐1 methylation showed a negative correlation with IL‐6 and a positive correlation with iNOS methylation.^40^ In contrast, a relatively recent EWAS (Project VIVA) revealed multiple differentially methylated CpGs and DMRs for various parameters including FeNO, allergic and current asthma, allergen sensitization, AR, and lung function with the largest number of differentially methylated sites and DMRs to be associated with FeNO levels. Discovered DMRs annotated to genes implicated in allergy and asthma, Th2 activation and eosinophilia (EPX, IL4, IL13, PRG2, CLC and ZFMP1) and genes previously associated with asthma and IgE in an EWAS of blood (ACOT7, SLC25A25). Solute carriers and intracellular transport genes such as SLC25A25, SLC39A4, DNAH17 and VTI1A were differentially methylated among allergic asthmatics, and were specifically associated with FeNO. Other observed differential nasal DNAm of asthma‐associated genes for FeNO was associated with T‐cell activation, oxidative stress, and mucin production. Hypomethylation of a gap junction protein gene (GJA4) was observed for sensitization to environmental allergens,^13^ and lower DNAm of several CpGs in genes regulating eosinophilic and Th2 responses was associated with FeNO and allergic asthma. Greater DNAm of several CpGs annotated to the PRTN3 gene, levels of which have previously been shown to differ in the nasal epithelium of individuals with current AR,^56^ was associated with higher FeNO and allergic asthma. Differential methylation of IL‐4 and IL‐17 signaling pathways appeared to be affected by FeNO in asthma, as well as allergic asthma.^13^ The replication external cohort for allergic asthma confirmed 61% differentially methylated CpGs (58 CpGs), including several sites annotated to the ACOT7, ZFPM1, PRG2, EPX and EVL genes. Of note, multiple DMRs (EPX, ACOT7 and SORCS2 genes) were overlapping across phenotypes like FeNO, allergic asthma, environmental IgE sensitization, and total IgE. Bronchodilator response (BDR) was the only trait with a unique nasal DNAm signature, 130 distinct CpGs, showing no overlap with any of the other phenotypes considered.

Some years earlier, in 2017, Yang et al. (Inner City Consortium)^47^ was the first to reveal atopic asthma‐specific DNA methylation in nasal epithelial cells from allergic asthmatics (*n* = 36) when compared to age‐matched healthy subjects. In particular, they identified 119 genome‐wide significant DMRs associated with 118 unique genes and 118 single CpG motifs (DMPs) associated with 107 unique genes. The median percent methylation difference between allergic asthmatics and controls was quite high (6.8% for DMRs and 13.6% for DMPs). Among the 186 (DMR and/or DMP) differentially methylated genes associated to allergic asthma in the nasal epithelium, there are genes with established roles in asthma and atopy, immunity, cell adhesion, epigenetic regulation, airway obstruction, obesity and autophagy.

Another large cohort study (EVA‐PR) showed that 8664 CpGs were differentially methylated by atopy, with some of the most significant CpGs annotated to genes including *CDHR3, CDH26*, SLC9A3, PCSK6, FBXL7 and NTRK1, which are biologically plausible candidate genes for atopy. 1570 CpG‐gene expression pairs were revealed, including 11 of the top 30 EWAS results.^48^ An enrichment analysis revealed that the methylation signals linked to atopy or atopic asthma are in pathways related to gap junction signaling and immune regulation, including antigen presentation and Th1/Th2 signaling. Several CpG/gene pairs showed significant mediation, including SLC9A3, CDH26, PCSK6 and MAP3K14; others, such as FBXL7 or NTRK1, showed little or non‐significant mediation, suggesting that the link between methylation and atopy occurs through other mechanisms.^48^

Distinct nasal epithelial DNAm were also observed between non‐severeand severe asthma in African‐American children and may be useful in predicting disease severity. Several of the annotated genes, play a critical role in asthma.^57^, ^58^, ^59^, ^60^, ^61^, ^62^ Six DMPS, which revealed to be associated with asthma severity, annotated to *TMEM51*, *WDR25*, *HIPK3*, and *KLF11*,^49^ were associated with clinical features of asthma in Project VIVA,^13^ supporting the involvement of these identified CpGs in regulation of asthma severity. DNAm levels of 39 DMPs significantly correlated with mRNA levels in children with RNA‐seq data available. Moreover, enrichment was observed for three regulatory histone marks associated with functional gene regulatory elements around CpG sites associated with asthma severity,^49^ confirming the hypothesis that histone modifications are essential for the pathogenesis and progression of asthma, which regulates gene function together with, or independent, of DNAm.^63^, ^64^

Regarding AR, data indicating its association with the methylation of nasal epithelial cells is scarce. To the best of our knowledge only two independent cohorts have been conducted to explore the differences in nasal DNAm profiles between people with or without rhinitis. Qi et al. recruited 455 cases with asthma or rhinitis or a combined asthma/rhinitis phenotype to uncover possible shared epigenetic associations (PIAMA cohort). In total, 81 CpG sites were significantly associated with rhinitis and 75 were associated with the combined asthma/rhinitis phenotype (AsRh) while most of them were replicated in the EVA‐PR cohort. A total of eight CpG sites in nasal epithelium showed association with all three phenotypes, five of which are near known biologically plausible genes related to allergic disease, including NCF2, which is involved in the oxidative stress pathway and related to asthma^24^; NTRK1, an epigenetic target of IL‐13 involved in allergic inflammation GJA4, the expression of which has been associated with airway inflammation and bronchial hyperresponsiveness^65^; CYP27B1, an enzyme, the activity of which has been associated with IgE‐dependent mast cell activation^66^; and ANO1 (anoctamin 1), which is related to chloride conductance in airway epithelial cells and is upregulated in epithelial cells of patients with asthma.^50^ On the other hand, the study by Cardenas et al.^13^ concluded in no associations between nasal DNAm and AR. Nevertheless, the important role of bacterial colonization of the upper airways during early life (1 week), where the children with AR showed lower richness in nasal microbiota compared to children without AR, has been proved in shaping epigenetics profiles of nasal epithelium that persist at least to later childhood (6 years), and contribute as a significant factor to the development of AR.^67^

The epigenome is determined by the genome and genetic variation and epigenetic variation influence each other.^68^ Several CpGs in the human genome are implicated in Methylation Quantitative Trait Loci (mQTLs), that is, the methylation level is at least partly determined by genetic variants in cis or in trans. However, the proportion of the variance in the methylation levels explained by genetic variation is in most cases rather limited.^69^ Studies analyzing epigenetic and genetic variation at large scale in the same individuals are so far limited in allergic diseases. Evidence from GWAS has shown that SNPs are not always associated with expression of nearby genes, but rather that of more distant cis‐genes within 1 Mb. While genotype does not change as disease progresses, both epigenetic regulation and transcriptomic activity change as the disease develops or worsens. Thus, studying eQTM (genome‐wide expression quantitative trait methylation) may complement findings from genetic or eQTL studies and add novel insights into asthma/rhinitis pathogenesis.

DNA methylation affects gene expression; thus, Qi et al. examined whether DNA methylation was associated with local gene expression by *cis*‐eQTM analyses. Association was found for 24 of the 68 investigated CpG sites. The most significant negative association was cg18297196‐TREM1. TREM1‐associated neutrophilic signaling pathway proteins have been reported to be significantly suppressed in eosinophilic nasal polyps of patients with chronic rhinosinusitis.^70^ A total of 20 CpG site–gene pairs showed significant association between CpG sites and genes where the CpG sites were located, including PCSK6 (proprotein convertase subtilisin/kexin type 6), FBXL7 (F‐box and leucine rich repeat protein 7) and CISH (cytokine inducible SH2 containing protein). PCSK6 (NFkb pathway activato‐r)^71^ FBXL2 (involved in inflammation), FBXL7 (involved in corticosteroid response^72^; and CISH (increased in allergen challenge^73^ are genes associated with allergy or inflammation. Genes identified by eQTM were enriched in pathways related to immune functions and inflammatory responses.^50^ Another eQTM analysis in nasal airway epithelium was conducted by Kim et al. 2020 including subjects from the EVA‐PR study. The top 500 eQTM genes were enriched in pathways for immune processes and epithelial integrity, and more likely to have been previously identified as differentially expressed in atopic asthma. Most of the top eQTM genes have been implicated in lung disease for example *PAX8*; associated with bronchodilator response in children with asthma, *ECHDC3* with obesity and asthma in children, *LSP1* with acute lung inflammation, *HLA‐DQB1* with asthma and total IgE, *and KANSL1* with pulmonary function. Few of the most significant eQTM methylation‐gene pairs found in EVA‐PR cohort, also replicated in another publication, were STAT4, IL32, STAT1, CCR5, CCL5, HLA‐DMB, HLA‐DMA and CXCR6. Some of the 10‐transcription factor (TF) binding site motifs in enhancer regions associated with differentially methylated CpGs and differentially expressed genes (DEGs) in atopic asthma are FOXA, ALOX5 and GATA‐6.^51^

DNAm and corticosteroid treatment seems to present a bilateral relationship; acute systemic steroid treatment modifies nasal DNAm in good responders but nasal DNAm can differentiate response to treatment as well. Xiao et al. suggested that VNN1 as a biomarker for corticosteroid treatment response due to its altered methylation at the CpG site of the promoter. In particular, the methylation level at the CpG4 site trended to decrease in the poor responders, but increase in good responders following treatment. Moreover, there was a positive correlation between the change in DNA methylation at CpG4 (mT1–mT0) and VNN1 mRNA expression.^41^ An EWAS indicated that variation in DNAm levels was associated with treatment response, in particular 309 CpGs were identified whose DNAm levels were associated with treatment response—182 overlap with common SNPs including *LDHC*, *DNHD1* and *PRRC1* and 127 do not have SNP co‐localization including *GALNTL4*, *SRD5A3*, *MED12L‐GPR87* locus and *CARD14.* Similarly, systemic steroid treatment significantly altered the nasal methylome within 24 h in good responders; specifically, the promoter of *OTX2* showed significantly decreased methylation. The *OTX2* gene encodes a transcription factor with key roles in the brain^74^, ^75^ craniofacial and sensory organs^76^, ^77^ and pituitary development,^78^ directly interact with Foxa2,^79^, ^80^ which has been implicated in suppression of goblet cell metaplasia during allergen challenge and the production of IL13, IL33, CCL20 and CCL17 from airway epithelial cells and inhibits allergen‐induced goblet cell differentiation,^81^ through reprogramming of Th2‐mediated inflammation and innate immunity.^42^ Contrary Yang et al.^47^ showed no DMRs/DMPs association with nasal corticosteroid use among asthmatics, after adjustment for multiple comparisons; thus, the relationship of corticosteroid treatment and DNAm remains to be clarified.

Potential causes of methylation changes include pet exposure, cigarette smoke,^82^ air pollution^83^ and farming aerosols^84^ which affect the epigenome, while pet exposure at early life has also been implicated.^85^, ^86^, ^87^ Pet exposure at secondary school age has been positively associated with current nasal methylation levels of cg03565274 annotated to ZMYND10 (a gene related to related to primary ciliary dyskinesia).^88^ The methylation level of the later is negatively associated with a compared asthma‐rhinitis phenotype (AsRh) suggesting that some environmental exposures could affect DNA methylation in the nasal epithelium, which may have protective effects on AsRh. Methylation‐related expression of ZMYND10 in AsRh is lower in nasal epithelial cells, or alternatively, it may be explained by a lower subset of differentiated ciliated cells in AsRh compared with healthy controls, as was recently discovered in patients with chronic rhinosinusitis through use of scRNAseq.^89^ The influence of environmental exposures on the nasal epithelial epigenome was highlighted by identifying 48 DMRs in 46 unique genes that are significantly associated with environmental tobacco smoke.^47^ Nevertheless, regarding AsRh, no significant associations have been shown with exposures to other potential risk factors for allergic disease, such as smoking, secondhand smoking, moulds and dampness.^50^

Μicrobial species are also known to influence the epigenome,^82^, ^83^, ^84^ for instance, respiratory virus infections affect NECs DNA methylation. Analysis of Alu methylation indicated increased global methylation occurred in NECs of asthmatics in response to virus infection.^43^ The ‘Inner City’ genome‐wide methylation analysis after rhinovirus (RV) infection identified 27,517 CpGs differentially methylated, classified into 11 different functional groups, of which 10,498 was increased, while 15,155 CpGs showed a decrease when asthma was compared with controls.^44^ DNA methylation profiles in response to infection in NECs from both healthy and asthmatic subjects were not dominated by loci associated with proinflammatory or antiviral immune responses; a principal component analysis pointed to a common locus where SNORA12 is located. While the function of SNORA12 in HRV infection remains to be determined, changes in SNORA12 gene expression during virus infection have been observed. Despite being common in response to infection, SNORA12 methylation differed in atopic asthmatic and healthy subjects and a relationship between SNORA12 gene expression and lung function within the different groups was observed, implying that methylation‐mediated changes in gene expression caused by underlying respiratory disease may also have functional consequences.^43^ Another study revealed 471 CpGs significantly different between asthmatics and controls, comprising in total 268 genes to have HRVI‐induced asthma‐specifically modified DNA methylation and mRNA expression. Of these, 16 (AGPAT1, BAT3, NEU1, ANAPC11, MGST3, CCT6A, MICB, SMN1, LOC442454, KLHL8, SLC16A3, GP1BA, DNAJC7, VDAC2, FBXO7 and TP53I3) showed a change in DNA methylation of greater than 3% which were accompanied by an mRNA expression change greater than 0.1 RPKMS (Reads per kilo base per million mapped reads).^44^ Some of them such as *BAT3* and *NEU1* have been previously investigated in the context of asthma and HRVI. HRVI‐induced *BAT3* expression was reduced in children with asthma compared to children without asthma. *BAT3*, also known as HLA‐B‐associated transcript 6 (*BAG6*), encodes for a cytoplasmic protein which is involved in mammalian cell apoptosis and proliferation^90^, ^91^ and in the activation process of natural killer cells initiating IFN‐γ and TNF‐α cytokine release.^92^, ^93^ The catalytic enzyme Neuraminidase 1 *(NEU1)* plays a role in the T helper type 2 (Th2)‐mediated airway inflammation in a murine acute asthma model^94^ and forms complexes with Toll Like Receptors (TLR2, 3, 4) activating TLR signaling.^95^, ^96^

## 5 DNA METHYLATION IN BLOOD

Methylation status in either whole blood or PBMCs and the linkage to asthma has been widely studied^97^, ^98^, ^99^ and has been extensively discussed in a plethora of reviews; however, only few studies discriminate allergic asthma and atopy. There is a significant interaction effect among global DNA methylation levels in blood, asthma severity, race/ethnicity, and socioeconomic status; particularly African American children present higher levels of global DNA methylation than children of other races/ethnicities.^100^ Apart from race/ethnicity, gender‐specificity of DNAm changes have been recorded and have been associated with asthma acquisition from pre‐adolescence to late‐ or post‐adolescence in birth cohorts. A particular subset of CpGs has been linked with male gender while other loci are affecting mostly women, without a specific gender direction; nevertheless, CpGs are likely to play a role in the underlying mechanisms of sex‐specific asthma acquisition.^101^ DNA methylation levels in PBMCs significantly differ between children with IgE sensitization compared to non‐sensitized children including genes related with the mTOR signaling pathway and the MAP kinase pathways. Of note, the differences in DNA methylation associated with IgE sensitized children at 5 years of age can be detected already in maternal PBMCs, cord blood, and at age 2Y making them as potential candidate for biomarker.^102^ In general, allergic subjects present mainly a decreased DNAm and the CpGs are annotated to genes with biological functions relevant to allergic sensitization such as the regulation of ILs production.^103^ In a large EWAS from the MEDALL consortium using four European birth cohorts and further seven validated cohorts, childhood asthma was found to be associated with a number of differentially methylated CpG positions in whole blood.^104^

Nevertheless, the vast majority of the large cohorts (ewas studies) involve either whole blood or PBMCs while only a few also included blood cell populations that are sorted in limited size cohorts. Of note, up to 40% of the differences in methylation profiles of individuals could be attributed to the cell‐type heterogeneity of white blood cells.^105^ The best studied type of blood cells is eosinophils; purified circulating eosinophils showed an altered DNA methylation profile, usually hypomethylation, suggesting a differential activation state which affect immune functions of this subpopulation, the interaction with other PBMCs and certain lung functions.^98^, ^104^ Genes implicated in airway remodeling, surfactant secretion and nitric oxide production in airways, as well as genes associated with cytokine production and signaling and phagocytosis in blood are characterized by decreased methylation in asthmatic subjects.^106^

Four recent large epigenome wide association studies have been conducted concerning the relationship of atopy and DNA methylation. Distinct methylation signals are found between non‐atopic and atopic asthma as well as between atopic and healthy subjects. In Agricultural Lung Health Study, several hundred CpG sites were differentially methylated in blood DNA from adults with non‐atopic or atopic asthma compared to adults with neither asthma nor atopy while 99.5% of these CpG sites presented decreased methylation. Atopic asthma CpG sites were enriched in pathways involved in inflammatory response or characterized by chronic inflammation consistent with the inflammatory nature of atopic asthma.^107^

Differential methylation has been revealed in peripheral blood associated with atopic sensitization, environmental and food allergen sensitizations (Project Viva/Generation R Study^108^ and IoW study^103^ during mid‐childhood and adolescence, respectively; methylation sites were annotated to genes that have been implicated in asthma pathway and with biological functions relevant to allergicsensitization, mTOR signaling, inositol phosphate metabolism and the regulation of IL‐5 production.^103^, ^108^ Although studies suggest that epigenetic marks in cord blood DNA may serve as early biomarkers of allergic susceptibility in childhood as some of these CpGs had nominal associations with cord blood and genes involved in airway remodeling, there are not enough data to support this suggestion.^108^

The cohort of Wu et al.^109^ (MeDALL study) revealed 80 CpGs differentially methylated in whole blood to be associated with allergy in the discovery phase but after meta‐analysis of the results from 8 different cohorts, they concluded in 21 decreased methylated CpG sites. Contrary to the findings of Acevedo et al.^102^ and Peng et al.^108^ DNA methylation at these 21 CpG sites in cord blood was not predictive of allergy during early childhood, indicating that postnatal changes in DNA methylation may play an important role in the development of allergy. Correlation of these 21 CpG sites with genome‐wide gene expression revealed clusters of genes linked to airway inflammation, pathogenesis of lung diseases, development of immune responses, genes correlated with blood eosinophil counts, activation of granulocyte production and genes associated to asthma.^109^

## 6 DNA METHYLATION COMPARISONS AMONG STUDIES AND ACROSS TISSUES

**FIGURE 1:**
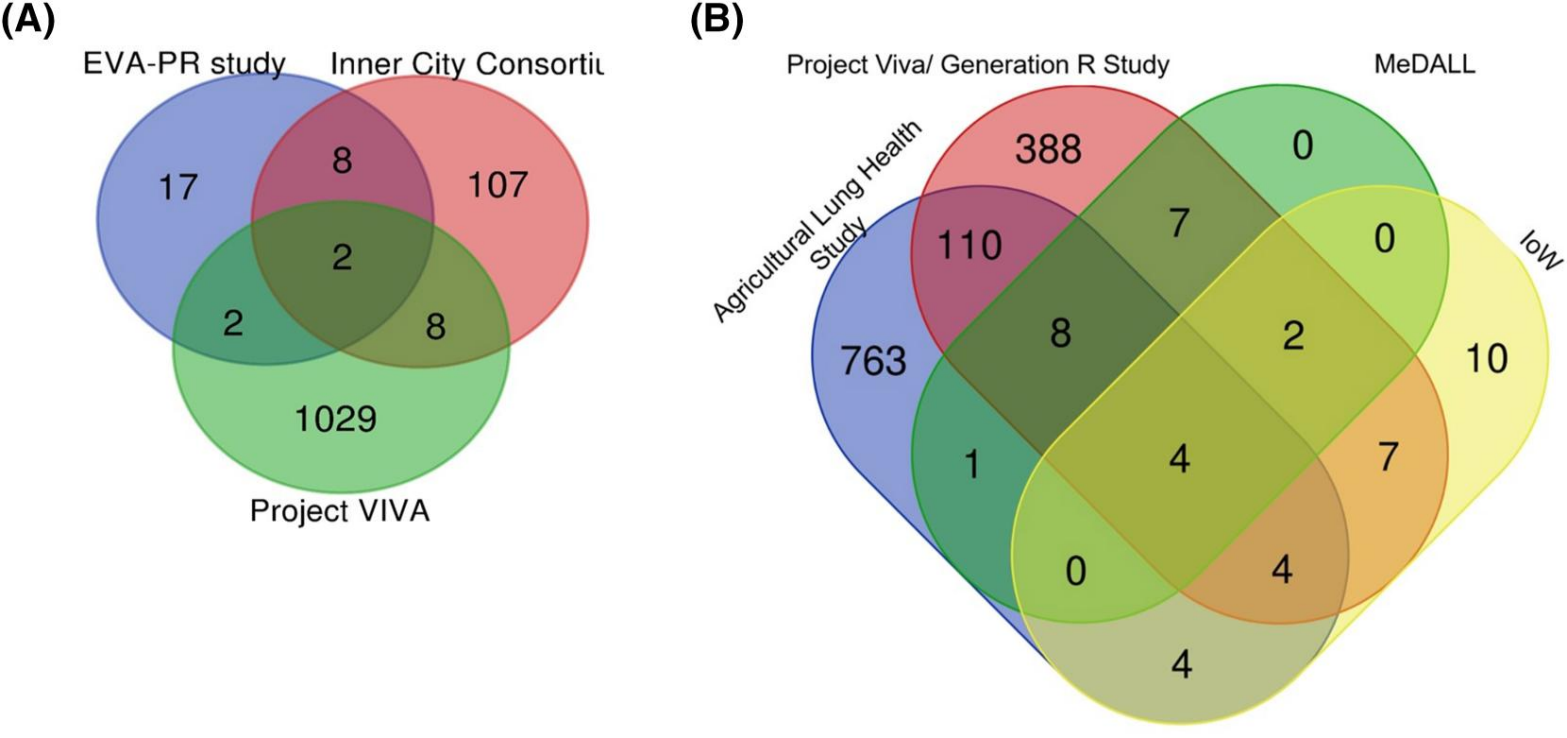
(A) Venn diagram representing the shared differentially methylated gene loci associated with atopic asthma in nasal epithelium samples among three large EWAS: Project Viva,^13^ Inner City Consortium,^47^ EVA PR study.^48^ B, Venn diagram representing the shared differentially methylated gene loci associated with allergic sensitization and allergic asthma in whole blood samples among four large EWAS

Our comparison of the aforementioned EWAS brings up some important gene loci that overlap in each tissue (Figure 1A,B). For instance, the comparison of the four EWAS in whole blood samples^5^, ^107^, ^110^, ^111^ highlights the following genes: KCNH2, ACOT7, EPX, NHLRC4 (Table S1). When we compared the three EWAS in NECs (EVA‐PR study, Inner City Consortium and Project VIVA; Table S2), after considering only the methylation status of allergic/atopic asthma and including all the presented genes with statistical significance, two shared gene loci are identified: PCSK6 and TSHR. PSCK6 has also been differentially expressed in bronchial brushings between cigarette smoking severe asthma and nonsmoking severe asthma^111^ and has been mentioned to be induced in vitro by interleukin (IL)‐4 and IL‐13, to activate NF‐κB, IL‐1, and IL‐6,^25^ to have a paracrine role in activating matrix metalloproteases^30^ and participate in glycoprotein metabolic process and endopeptidase inhibitor activity. Giovannini‐Chami et al.^110^ included PSCK6 among the 61 most discriminant genes between dust mite AR and healthy children. TSHR encodes a hormone ligand‐binding receptor, known for its role in autoimmune thyroid disease which participate in mediating anti‐inflammatory cytokine production as well as cAMP and G‐protein signaling pathways. TSHR does not have a well‐established association to asthma and/or atopy, but human fibrocytes, whose increased number is implicated in asthma with chronic airflow obstruction, express the thyrotropin receptor. TSHR‐engaged fibrocytes generate extremely high levels of several inflammatory cytokines. This is a gene that warrants further exploration in the context of respiratory allergy.

There are several other DMRs in allergic asthma that appeared at least in two of the NECs studies (Table S2). Cardenas et al.^13^ comparing their results with ‘Inner City Consortium’ and ‘EVA‐PR’ studies indicated 53% and 61% of replicated CpGs respectively, all with consistent direction of association including multiple CpGs annotated to EVL and EPX genes. Notably, several of the top differentially methylated CpGs revealed by Cardenas et al.^13^ were shown to be associated with the expression results of EVA‐PR including METTL1 for environment IgE sensitization, NTRK1 for allergic asthma, and several genes, such as MAP3K14, NTRK1, FBXL7, PCSK6, SLC9A3, CDH26, CAPN14 and MAP3K14 for FeNO. Pech et al.^44^ suggested that 14 shared asthma‐specific CpGs (annotated to the following genes: LDLRAD3, METTL1, CDC45, C15orf54, DUOX1, ZFPM1, LDLRAD3, ALOX15, POSTN and CTCS) after a comparison of ‘Inner City Consortium’ and their study ‘ALLIANCE’.

When considering the same type of tissue, several common differentially methylated loci have been revealed. Of importance is also whether methylation differences are established across tissues. The first indication comes from a Project VIVA comparison where 13/14 asthma associated CpG sites tested in whole blood^104^ were found also differentially methylated in nasal cells samples.^13^ On the other hand, nasal DNAm findings presented a minimal overlap with cord blood DNAm patterns prospectively linked to development of childhood asthma and allergy. The comparison between data of complete remission versus persistent asthma from whole blood samples^112^ and from bronchial biopsies^29^ indicated one common CpG (cg13525448), annotated to *LBX1* and *TLX1* genes to have the same direction of effect. This consistency suggests that the methylome signature in asthma can be stable in whole blood, nasal cells, and potentially other tissues. Contrary, some other genes, that GWAS and EWAS in nasal samples have correlated to asthma, did not show a similar methylation pattern in whole blood samples suggesting that the nasal cellular compartment may be more sensitive to asthma‐associated DNAm differences, and potentially an optimal tissue for detecting epigenetic modifications of known biological relevance.^13^

**FIGURE 2:**
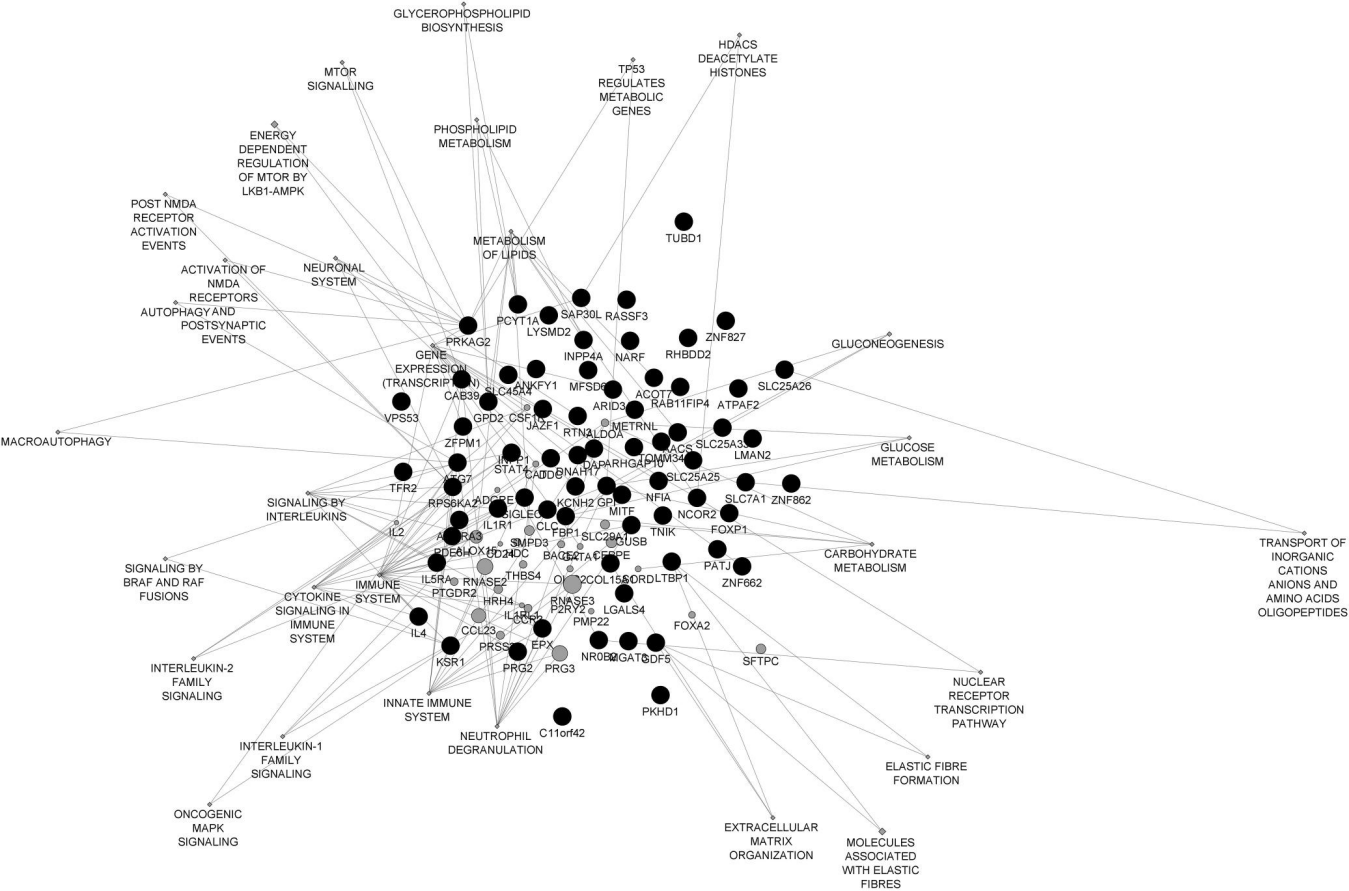
Cytoscape diagram presenting the network of common differentially methylated genes in at least three studies and the enriched pathways are implicated using reactome‐pathway analysis tool

However, the question remains whether there are differentially methylated loci annotated to common genes across the various tissues in allergic asthma. A comparison of genes to which significant CpGs and DMR were annotated from available EWAS dealing with allergy and allergic asthma reveals a few common genes in whole blood and nasal epithelium samples but no shared genes with bronchial epithelium samples. The inconsistency of results between the studies could be attributed to the great variability in age and gender distribution between studies, as well as clinical features such as disease's definition and severity and other factors such as smoking. Thirteen differentially methylated loci were presented in at least four studies including both blood and nasal samples making them possible candidates for biomarkers (Table S3). A Reactome‐pathway analysis of these genes, demonstrated at a cytoscape diagram (Figure 2), revealed the most relevant significantly enriched pathways) including pathways involved in immune system, cytokine signaling and metabolic procedures (gluconeogenesis, glycose‐lipid‐carbohydrate metabolism) The results of this comparison are in agreement with the results of a meta‐analysis by Reese et al.^98^ which correlated ACOT7, EPX, KNHC2, ZNF862, IL5RA and ZFPM1 with childhood asthma. Of note, the ACOT7 gene was present in 6/8 compared studies including whole blood and nasal samples. Considering this gene, its methylation status has also been significantly associated with total IgE.^113^ A life course analysis of IgE hypersensitivity studying the change in methylation between cord blood and mid‐childhood DNA revealed significant postnatal differentially methylated sites located within ACOT7 (four sites) and ZNF862 (three sites).^114^ EPX plays a role in various immune system pathways such as the defense to microorganisms, regulation of ILs, response to oxidative stress and neutrophil degranulation. The association of EPX levels with asthma is well studied; both urinary and serum EPX levels have been linked to childhood asthma and have been suggested in the past as biomarkers.^115^ Concerning IL5RA, there are plenty of data linking it with asthma in a positively correlating manner. Moreover, polymorphisms in *IL5RA* account as a genetic risk factors for asthma development, especially in atopic populations.^116^, ^117^ Furthermore, its function in inflammatory response, signaling transduction, like the cytokine mediated signaling pathway and MAPK cascade is known; thus, it consists a biologically plausible candidate marker for asthma development.

Unfortunately, EWAS studies that involve bronchial epithelial samples, the kind of tissue that is ‘closer’ to asthma, comparing methylated gene loci between healthy individuals and allergic asthmatics are scarce. In our comparisons, only one study^23^ evaluating the genome‐wide methylation profile of the bronchial mucosa of allergic asthmatics compared to non‐allergic was included, which is apparently not sufficient.

## 7 CONCLUSIONS

In conclusion, epigenomic and transcriptomic profiling has provided a means of exploring how gene‐environment interactions contribute to the pathology of asthma and other allergic respiratory conditions. Several genes and CpGs have been suggested as asthma biomarkers, though research is currently lacking on AR. In both blood and nasal samples, the most presented differentially methylated loci were annotated to ACOT7, EPX, KCNH2, SIGLEC8, TNIK, FOXP1, ATPAF2, ZNF862, ADORA3, ARID3A, IL5RA, METRNL, ZFPM1, making them possible candidates for allergic respiratory disease biomarkers. Overall, there is significant heterogeneity among studies, in respect to sample sizes, age groups, disease phenotype, statistical methods; however, the field appears to be very conscious of limiting factors, and a steady improvement is certainly evident over time in the quality and scope of newer studies.

BECs are not easily available but more clinically relevant to airway diseases compared to other types; therefore, more EWAS studies on BECs, including comparisons between healthy individuals and atopic asthmatics, are required for more precise associations. Distinguishing between atopic and non‐atopic asthma should be taken under consideration in future studies, as epigenetic differences between the two groups can be striking. In general, accumulated evidence suggests that NECs are comparable in diagnostic performance to BECs. However, it should be noted that most epigenetic studies were conducted on pediatric populations of varying age, with very few cases of older subjects. Studying DNA methylation differences in blood samples is technically easier. Nevertheless, their results may be less informative, regarding methylation marks in airway, despite some identified shared signals with NECs, that indicate the presence of cross‐tissue epigenetic mechanisms, which overlap both at the pathway and gene level.

Importantly, DNA methylation profiles were different depending on sample isolation techniques, which should therefore be strongly considered in future research. Only few studies assessed cell type composition, even though this variation can affect the results. Single cell RRBS is a promising method to assess the role of cell types, since the current approaches average out results, and do not consider the contribution of differences in individual cells. Additionally, a lack of variability in the analysis method has been noticed; thus, the combination of different methodologies, such as RRBS and WGBS, may be beneficial for reproducible conclusions and for the evaluation of new biomarkers.

Finally, it is essential to focus on more detailed investigation processes at all levels, in order to identify targeted methylation loci as potential biomarkers. The clinical characterisation of patients, the sampling procedure, the choice of technique and the analysis protocol play a role in DNA methylation results variability and repeatability. Wider studies in age groups with specific clinical characteristics and using more precise protocols will elucidate differentially methylated loci that could be used as immune age biomarkers in the future.

## CONFLICT OF INTEREST

We have no conflict of interest to declare.

## AUTHOR CONTRIBUTIONS

**Evangelia Legaki:** Investigation; methodology; validation; writing – original draft; writing – review & editing. **Christos Arsenis:** Investigation; writing – original draft; writing – review & editing. **Styliani Taka:** Conceptualization; supervision; validation; writing – review & editing. **Nikolaos G. Papadopoulos:** Conceptualization; resources; supervision; writing – review & editing.

## Supporting information

Supporting Information S1

## Data Availability

Data derived from public domain resources.
