# Supplementary material for: DNA methylation biomarkers in asthma and rhinitis: Are we there yet?
Source: Clin Transl Allergy. 2022 Mar 11;12(3):e12131. doi: 10.1002/clt2.12131 (PMC8967268; doi:10.1002/clt2.12131)
Supplement: Supplementary file 1 — Supporting Information S1 [file CLT2-12-e12131-s001.docx]

Table S1. Differentially methylated gene loci associated to allergic sensitization and allergic asthma in four whole blood- studies.

| **Study** | **Number of common genes** | **Genes** |
| --- | --- | --- |
| Agricultural Lung Health Study ([107](#_ENREF_107)), MeDALL study ([109](#_ENREF_109)), Project Viva /Generation R study ([108](#_ENREF_108)) IoW study ([103](#_ENREF_103)) | 4 | KCNH2 ACOT7 EPX NHLRC4 |
| Agricultural Lung Health Study ([107](#_ENREF_107)), MeDALL study ([109](#_ENREF_109)), Project Viva/ Generation R study ([108](#_ENREF_108)) | 8 | TNIK SIGLEC8 MED27 SLC2A8 SMURF1 FOXP1 LPCAT3 ATPAF2 |
| Agricultural Lung Health Study ([107](#_ENREF_107)), Project Viva/ Generation R study ([108](#_ENREF_108)) IoW study ([103](#_ENREF_103)) | 4 | SEC16B IL5RA FADD METRNL |
| MeDALL study ([109](#_ENREF_109)),  Project Viva/ Generation R study ([108](#_ENREF_108)) IoW study ([103](#_ENREF_103)) | 2 | ZFPM1 FAM53B |
| Agricultural Lung Health Study ([107](#_ENREF_107)), Project Viva/ Generation R study ([108](#_ENREF_108)) | 110 | AACS TUBD1 LPP C11orf42 RASSF3 PROZ AGPAT1 MFSD6 NFIA SLC25A26 CLC CALML4 LTBP4 ABR DICER1 A2ML1 COL15A1 YWHAQ RTN3 ARID3A DENND1A PIK3CB LRRC17 TOMM34 GPI TFR2 STAT4 LTBP1 SETMAR RD3 LYSMD2 ATP2C1 RCC2 GTSF1L CAMSAP1 CATSPER4 BRD4 KSR1 ASAP1 RHBDD2 LPCAT2 ZNF827 PDE6H OSBPL3 LOC728989 LGALS4 RAB11FIP4 DPPA3 TFF2 TRANK1 SLC19A1 ENPP2 VPS53 PRKAG2 JAZF1 WWOX ARHGAP10 FOXK1 RPS6KA2 ZNF862 SLC25A25 ENG FAM20B GAPVD1 KLF1 SAP30L LMAN2 PRKCZ NARF ALOX5AP MND1 KCNAB2 CAPN2 RNASE7 MYT1L ANKFY1 CREG1 IL4 PRG2 ADORA3 ZFYVE28 GUSB EXD3 SLC25A33 FBP1 SLC45A4 MCC DDC GDF5 C19orf54 CAB39 NR0B2 PDE4B LRP12 IRGC ZC3H11A SEPT8 SLC7A1 GPD2 MGAT3 DAP TTC17 INPP4A ZNF662 CEBPB TFF1 STON1-GTF2A1L TMOD3 ITGAL ZNF22 |
| Agricultural Lung Health Study ([107](#_ENREF_107)), MeDALL study ([109](#_ENREF_109)), | 1 | PATJ |
| Agricultural Lung Health Study ([107](#_ENREF_107)),  IoW study ([103](#_ENREF_103)) | 4 | MITF DNAH17 WEE1 MFHAS1 |
| MeDALL study ([109](#_ENREF_109)),  Project Viva/ Generation R study ([108](#_ENREF_108)) | 7 | BANK1 NDFIP2 TTC7A GUCA2A CYB5B CLU LOC339524 |
| Project Viva/ Generation R study ([108](#_ENREF_108)) IoW study ([103](#_ENREF_103)) | 7 | PCYT1A ASCC1 FAM81B C8orf47 DST B4GALT7 C13orf35 |

Table S2. Differentially methylated gene loci associated to atopic asthma in three nasal epithelium EWAS studies.

| **Study** | **Number of common genes** | **Common Genes** |
| --- | --- | --- |
| EVA-PR study ([51](#_ENREF_49)),  Inner City Consortium ([42](#_ENREF_48)), Project VIVA ([13](#_ENREF_13)) | 2 | PCSK6 TSHR |
| EVA-PR study ([51](#_ENREF_49)), Inner City Consortium ([42](#_ENREF_48)) | 8 | CAPN14 GJA4 C22orf31 EFNA5 FBXL7 SPP2 CES4A METTL1 |
| EVA-PR study ([51](#_ENREF_49)),  Project VIVA ([13](#_ENREF_13)) | 2 | PDE6A NTRK1 |
| Inner City Consortium ([42](#_ENREF_48)), Project VIVA ([13](#_ENREF_13)) | 8 | NCOR2 ARID3A ACOT7 NFIC ZNF862 PTPRC ADORA3 FAXDC2 |

Table S3. Comparison of the altered methylation findings of 8 different studies, including whole blood, nasal epithelium and bronchial epithelium samples

| **Study** | **Number of studies** | **Number of common genes** | **Common Genes** |
| --- | --- | --- | --- |
| Agricultural Lung Health Study -Blood ([107](#_ENREF_107)) Project Viva/ Generation R study - Blood ([108](#_ENREF_108)) MeDALL study - Blood ([109](#_ENREF_109)) IoW study – Blood ([103](#_ENREF_103)) Inner City Consortium - Nasal ([42](#_ENREF_48)) Project VIVA- Nasal ([13](#_ENREF_13)) | 6 | 1 | ACOT7 |
| Agricultural Lung Health Study -Blood ([107](#_ENREF_107)) Project Viva/ Generation R study - Blood ([108](#_ENREF_108)) MeDALL study – Blood ([109](#_ENREF_109))  IoW study - Blood ([103](#_ENREF_103)) Project VIVA- Nasal ([13](#_ENREF_13)) | 5 | 2 | EPX KCNH2 |
| Agricultural Lung Health Study -Blood ([107](#_ENREF_107)) Project Viva/ Generation R study - Blood ([108](#_ENREF_108)) MeDALL study - Blood ([109](#_ENREF_109)) Project VIVA - Nasal ([13](#_ENREF_13)) | 4 | 4 | SIGLEC8 TNIK FOXP1 ATPAF2 |
| Agricultural Lung Health Study -Blood ([107](#_ENREF_107))  Project Viva/ Generation R study – Blood ([108](#_ENREF_108))  Inner City Consortium – Nasal ([42](#_ENREF_48))  Project VIVA - Nasal ([13](#_ENREF_13)) | 4 | 3 | ZNF862 ADORA3 ARID3A |
| Agricultural Lung Health Study -Blood ([107](#_ENREF_107)) Project Viva/ Generation R study - Blood ([108](#_ENREF_108)) IoW study - Blood ([103](#_ENREF_103)) Project VIVA - Nasal ([13](#_ENREF_13)) | 4 | 2 | IL5RA METRNL |
| Project Viva/ Generation R study - Blood ([108](#_ENREF_108)) MeDALL study - Blood ([109](#_ENREF_109)) IoW study - Blood ([103](#_ENREF_103)) Project VIVA - Nasal ([13](#_ENREF_13)) | 4 | 1 | ZFPM1 |
| Agricultural Lung Health Study -Blood ([107](#_ENREF_107))  Project Viva/ Generation R study – Blood ([108](#_ENREF_108))  Project VIVA - Nasal ([13](#_ENREF_13)) | 3 | 48 | AACS TUBD1 NFIA CLC TOMM34 TFR2 LTBP1 LYSMD2 ZNF827 PDE6H LGALS4 RAB11FIP4 VPS53 ARHGAP10 RPS6KA2 NARF IL4 PRG2 GUSB FBP1 NR0B2 SLC7A1 MGAT3 INPP4A C11orf42 RASSF3 MFSD6 SLC25A26 COL15A1 RTN3 GPI STAT4 KSR1 RHBDD2 PRKAG2 JAZF1 SLC25A25 SAP30L LMAN2 ANKFY1 SLC25A33 SLC45A4 DDC GDF5 CAB39 GPD2 DAP ZNF662 |
| Agricultural Lung Health Study -Blood ([107](#_ENREF_107)) MeDALL study - Blood ([109](#_ENREF_109)) Project VIVA - Nasal ([13](#_ENREF_13)) | 3 | 1 | PATJ |
| Agricultural Lung Health Study -Blood ([107](#_ENREF_107))  IoW study - Blood ([103](#_ENREF_103)) Project VIVA - Nasal ([13](#_ENREF_13)) | 3 | 2 | MITF DNAH17 |
| Project Viva/ Generation R study - Blood ([108](#_ENREF_108)) MeDALL study – Blood ([109](#_ENREF_109))  Project VIVA - Nasal ([13](#_ENREF_13)) | 3 | 1 | LOC339524 |
| Project Viva/ Generation R study - Blood ([108](#_ENREF_108)) IoW study - Blood ([103](#_ENREF_103)) Project VIVA - Nasal ([13](#_ENREF_13)) | 3 | 1 | PCYT1A |
| IoW study - Blood ([103](#_ENREF_103)) Project VIVA - Nasal ([13](#_ENREF_13)) Inner City Consortium – Nasal ([42](#_ENREF_48)) | 3 | 1 | NCOR2 |
| Bronchial study ([32](#_ENREF_32)) Project VIVA - Nasal ([13](#_ENREF_13)) | 2 | 3 | PKHD1 ATG7 IL1R1 |
